# Manioc anaphylaxis in a patient sensitised to latex

**DOI:** 10.1186/2045-7022-1-S1-P52

**Published:** 2011-08-12

**Authors:** Isabel Carrapatso, Borja Bartolome, Filipa Ribeiro, António Segorbe Luís

**Affiliations:** 1 Coimbra University Hospital, Imunoallergy Department, Coimbra, Portugal; 2 Bial-Aristegui, R&D Department, Bilbao, Spain

## Background

We report the case of a 51 years old female that suffered from a systemic anaphylactic episode 5 minutes after the ingestion of manioc at the age of 50. This patient reported rhinoconjunctivitis since she was 20 years-old after contact with latex gloves and rhinoconjunctivitis and oral allergy syndrome after the ingestion of kiwi, banana and mango since the age of 30. The patient had also a history of eight surgeries since the age of 24 years-old.

## Methods

Skin prick tests (SPT) to commercial extracts of aeroallergens and food allergens were carried out. Prick-to-prick tests (PP) and specific IgE determinations (sIgE) to some plant foods were also performed, according to case history. The molecular mass of the IgE binding bands was calculated by means of SDS PAGE immunoblotting. In order to study the presence of cross reacting IgE in the patient serum we carried out a SDS-PAGE immunoblotting-inhibition assay using manioc extract in solid phase and banana and latex as inhibitors. Furthermore, we carried out an immunoblotting inhibition assay using banana extract in solid phase and latex and manioc as inhibitors.

## Results

SPT and/or PP were positive to latex, manioc, chestnut, kiwi, tomato, banana and mango. Serum specific IgE was positive to latex (4.98 kU/L), Gold kiwi (0.4 kU/L) and banana 0.9 kU/L. The apparent molecular mass of the IgE binding bands on SDS PAGE immunoblotting assays were as follow: extract from mandioc pulp: 31 kDa; extract from latex: 55 kDa; 39 kDa; 34 kDa; 25 kDa; 20 kDa; 14 kDa; extract from Gold kiwi: 34 kDa; extract from banana: 34 kDa; 32 kDa . A total IgE binding inhibition on 31kDa manioc protein was detected when banana and latex extracts were used as inhibitors. When banana extract was used in solid phase a total IgE binding inhibition with latex was observed.

## Conclusions

Immunoblotting-inhibition assays proved a manioc-banana- latex cross reactive syndrome in this patient. We believe that, in this case, the primary sensitization agent is latex.

